# Vulcanization at Low Temperature: Its Advantages

**Published:** 1904-11

**Authors:** 


Vulcanization at Low Temperature: Its Advantages.—I think that the liability to disintegration or discoloration in pink, black, or red rubber may be somewhat obviated and that any kind will make a more perfect plate, if vulcanized for a long time at a low temperature, and I have found that pink rubber when treated in that way gives greater strength, durability and impenetrability. The practice of vulcanizing pink rubber or other rubber for the usual time of one hour at a temperature of 320 degrees is not a proper one; no less time than an hour and half at from 308 to 310 degrees, or far better still, three hours at 290 degrees, will give a much more satisfactory result. There is not a pink rubber upon the market but is increased in strength and durability just as the red rubber is, by vulcanizing a long time at a lower temperature. Pink rubber will absolutely not change one iota in color in ten, fifteen, or twenty years, if vulcanized three hours at a temperature of 280 degrees, running it up to 290 degrees, running it up to 290 degrees at the end of the third hour. I say that because I have proved it in my experience. It will not disintegrate and will not be penetrated by fluids of the mouth. So that you, who are wedded to the pink gums, if you treat your rubber in that way, will find that the color will be maintained, and the impenetrability will be just as good as that of the red rubber that you vulcanize now at 320 degrees for one hour.—*Western.*

---

It frequently appears that in an attempt to remove a perfectly healthy pulp after pressure anesthetizing and by the ordinary medium of entangling on a broach or nerve bristle, there may be observed a certain resilience of the tissue, so that it stretches like a rubber cord, springing back after breaking off. Here it is that trouble may supervene. I have noted in various instances more or less inflamation in the apical region following this stretching. In the case of one patient, a Yale College student, who came at the last hour to me, as his vacation drew to a close, a wholly unexpected complication set in after the removal of pulps under pressure in two bicuspids. The pulps were easily removed, leaving in this instance no occasion for returning sensitiveness. Yet both teeth troubled the patient during that night and despite the appearance of a favorable condition within the roots, no sensitiveness whatever appearing, I hesitated to fill, large contours being required, and in the end dismissed him to the care of a New York friend and fellow-practitioner.—*Era.*

